# Mitochondrial DNA enhance innate immune responses in neuromyelitis optica by monocyte recruitment and activation

**DOI:** 10.1038/s41598-020-70203-x

**Published:** 2020-08-06

**Authors:** Mikito Shimizu, Tatsusada Okuno, Makoto Kinoshita, Hisae Sumi, Harutoshi Fujimura, Kazuya Yamashita, Tomoyuki Sugimoto, Shuhei Sakakibara, Kaori Sakakibara, Toru Koda, Satoru Tada, Teruyuki Ishikura, Hisashi Murata, Shohei Beppu, Naoyuki Shiraishi, Yasuko Sugiyama, Yuji Nakatsuji, Atsushi Kumanogoh, Hideki Mochizuki

**Affiliations:** 1 Department of Neurology, Osaka University Graduate School of Medicine, 2-2, Yamadaoka, Suita, Osaka 565-0871 Japan; 2 Department of Neurology, Osaka-Toneyama National Medical Center, 5-1-1, Toneyama, Toyonaka, Osaka 560-8552 Japan; 3 Faculty of Data Science, Shiga University, 1-1-1, Baba, Hikone, Shiga 522-8522 Japan; 4 Laboratory of Immune Regulation, Immunology Frontier Research Center, Osaka University, 3-1, Yamadaoka, Suita, Osaka 565-0871 Japan; 5 Department of Neurology, Faculty of Medicine, University of Toyama, 2630, Sugitani, Toyama, 930-0194 Japan; 6 Department of Respiratory Medicine and Clinical Immunology, Osaka University Graduate School of Medicine, 2-2, Yamadaoka, Suita, Osaka 565-0871 Japan

**Keywords:** Autoimmunity, Neuroimmunology

## Abstract

Although recent studies indicate the involvement of monocytes in accelerating the lesion formation of neuromyelitis optica spectrum disorder (NMOSD), the precise mechanism of the innate immune system activation remains elusive. Thus, in this study, we aimed to clarify the mechanisms of NMOSD pathogenesis from the viewpoint of innate immunity activation. We established anti-AQP4 recombinant autoantibodies (Ab) from plasmablasts in NMOSD patient’s CSF. Human astrocytes treated with anti-AQP4 Ab produced a significant amount of CCL2 and contributed to the efficient recruitment of monocytes. Moreover, mitochondrial DNA (mtDNA), which activated monocytes via Toll-like receptor 9 (TLR9), was released from astrocytes treated with anti-AQP4 Ab. MtDNA further enhanced CCL2 production by monocytes, and it was demonstrated that mtDNA concentration correlated with the efficiency of monocyte recruitment in the CSF of NMOSD patients. In conclusion, these observations highlight that mtDNA which was released from astrocytes damaged by anti-AQP4 Ab has a central role in establishing the inflammatory loop of monocyte recruitment and activation via an innate immunity pathway.

## Introduction

Neuromyelitis optica spectrum disorder (NMOSD) is an inflammatory disease of the central nervous system (CNS) and anti-aquaporin4 autoantibodies (anti-AQP4 Ab) play a central role in its astrocytic injury^1–3^. The pathology of NMOSD is characterized by massive deposition of immunoglobulins and C9neo, or activated complement, predominantly at perivascular lesions^4^. Furthermore, numerous infiltrations of CD3^+^ and CD8^+^ T lymphocyte^3^ and more interestingly, in lesions of active demyelination^5^ and axonal loss, a massive infiltration of macrophages^3^ and intense activation of microglia^6^ are reported. These observations indicate that not only complement-dependent cytotoxicity (CDC) but also macrophage recruitment and activation induce the damage to oligodendrocytes and neurons.

Consistently, important roles of the innate immune cells, such as neutrophils and macrophage have been shown in recent studies of NMOSD disease models^7,8^. Intracerebral administration of NMOSD-IgG into naïve rats led to astrocytic damage in a CDC-dependent manner, and to recruite macrophages and neutrophils, resulting in myelin loss and neuron death. Importantly, the lesion size was reduced by macrophage depletion via clondronate in the model^7,8^. Thus, these observations suggest the pivotal role of monocyte recruitment and activation in accelerating the lesion formation of NMOSD.

However, the precise relationship between the astrocytic injury and monocyte involvement in NMOSD pathogenesis remains elusive. Recently, Yamashita et al. revealed that extracellular mitochondrial DNA (mtDNA) was increased in NMOSD patient CSF compared with multiple sclerosis (MS) and other neurological diseases, and the mtDNA released from astrocytes which AQP4-Ab damaged in a CDC-dependent manner enhanced microglia to secrete IL-1β via Toll-like receptor 9 (TLR9)^9^. Another report showed macrophages were activated and expressed NOD-like receptor family, pryin domain containing 3 (NLRP3) in NMOSD lesion^10^. Therefore, we hypothesized that mtDNA released from damaged astrocytes might serve as a bridge between acquired immunity cytotoxicity and innate immunity activation in NMOSD.

In this study, we aimed to reveal the pivotal role of mtDNA to recruit and activate peripheral monocytes in NMOSD pathogenesis.

## Results

### Pathogenic anti-AQP4 Abs derived from patient plasmablasts

**Figure 1:**
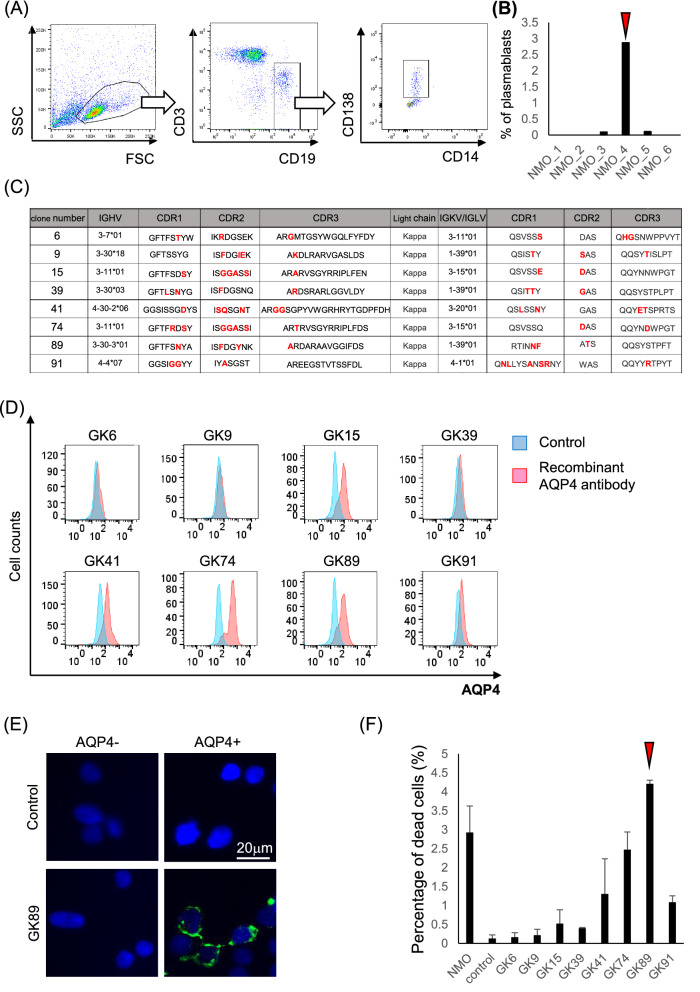
Generation of pathogenic anti-AQP4 recombinant antibodies derived from patients’ plasmablasts. **(A)** CD3^-^ CD19^int^ CD138^+^ plasmablasts are isolated from patients’ CSF lymphocytes. **(B)** The highest percentage of plasmablasts is observed in the sample derived from patient 4 (arrow head). **(C)** The interclonal diversity of V regions of eight clones which showed positive binding to AQP4 are shown in red. **(D)** Immunoreactivity to AQP4-expressing HEK293 cells is assessed by FACS analysis. The clones GK15, 41, 74 and 89 show remarkable binding to AQP4. **(E)** The immunoreactivity of clones GK89 to AQP4-expressing HEK293 cells is shown. **(F)** The percentage of damaged cells is measured by LDH assay. Clone GK89 has the highest capacity to induce complement-dependent cytotoxicity (arrow head). Error bars indicate SEM.

For the investigation of direct effects of anti-AQP4 Ab in the pathogenesis of NMOSD, we first aimed to establish pathogenic monoclonal AQP4 Ab derived from the patients. When we examined CSF plasmablasts characterized by CD3^−^ CD19^int^ CD138^+^ phenotype among six NMOSD patients (Fig. 1A), the CSF derived from patient 4 (arrow head) showed the highest percentage of CD3^-^ CD19^int^ CD138^+^ cells (Fig. 1B), and was further used for generating monoclonal AQP4 Ab by single-cell sorting. The DNA sequences of V regions of each clone showed interclonal diversity, represented by somatic hypermutations in the CDR regions of IgH, and either Igκ or Igλ (Fig. 1C).

Of the 29 clones generated, eight showed positive immunoreactivity to AQP4-expressing HEK293 cells in FACS analysis and immunohistochemistry (Fig. 1D and 1E). Of the eight clones, clones GK15, 41, 74 and 89 showed remarkable capacity to bind AQP4 (Fig. 1D). To identify the clone that possesses the highest capacity of CDC, LDH release was examined among eight clones utilizing AQP4-expressing HEK cells. The clone GK89 was the most potent to induce CDC in the LDH release assay (Fig. 1F) and was, thus, selected to be used in the following studies (arrow head).

### Chemokine signature of human astrocytes exposed to anti-AQP4 Ab

**Figure 2:**
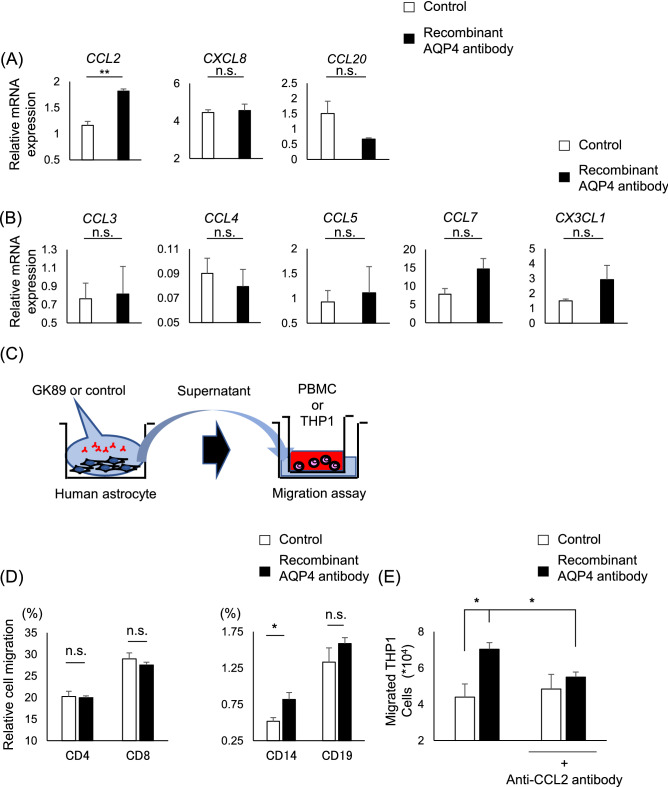
CCL2 released from human astrocyte has pivotal roles in monocyte migration. **(A, B)** Gene expression of chemokines in human astrocytes exposed to GK89. Values are normalized to β2-microgloblin or GAPDH. **(C)** The schematic view of migration assay utilizing supernatants of human astrocytes exposed to GK89 or control. **(D)** Supernatants of human astrocytes exposed to GK89 efficiently recruit CD14^+^ monocytes in comparison to CD4^+^, CD8^+^, and CD19^+^ cells. **(E)** The GK89-treated supernatant of human astrocytes significantly induces THP1 migration compared to the control group, and is efficiently inhibited by anti-CCL2 neutralizing antibody. Error bars indicate SEM. Three or more experiments are performed in each condition. *p < 0.05, **p < 0.01.

Human astrocytes exposed to GK89 showed significant upregulation of CCL2 (control; 1.16 ± 0.077, GK89; 1.82 ± 0.039, p = 0.0018) among potent chemokines which are known to be increased in NMOSD CSF, such as CXCL8 and CCL20 (Fig. 2A). Among the immune cells observed in inflammatory lesions, macrophages serve as an enhancer to form destructive lesion. Therefore, chemokines previously recognized to be involved in monocyte recruitment were further assessed. The expression levels of CCL3, CCL4, CCL5, CCL7, and CX3CL, however, did not show a significant difference between GK89-treated and control groups (Fig. 2B). These results suggest that human astrocytes targeted by anti-AQP4 Abs have the potentiality to attract monocytes by secreting CCL2.

### Monocyte recruitment by human astrocyte due to anti-AQP4 Ab exposure

As a significant release of CCL2 was observed in GK89-treated astrocytes, we examined whether human astrocytes exposed to anti-AQP4 Ab efficiently recruit monocytes by migration assay in vitro (Fig. 2C). The migration assay showed that among the migrating peripheral blood mononuclear cells (PBMCs), CD14^+^ monocytes (control; 0.52 ± 0.051%, GK89; 0.82 ± 0.093%, p = 0.049) were preferentially recruited by the GK89-treated astrocyte-conditioned medium (ACM) in comparison to CD4^+^, CD8^+^, and CD19^+^ cells (Fig. 2D). To elucidate the essential role of CCL2 for attracting monocytes by GK89-treated ACM, the migration of THP1 cells, human monocyte cell line, were investigated in the presence of anti-CCL2 neutralizing antibodies. The addition of anti-CCL2 neutralizing antibodies efficiently inhibited THP1 migration (Fig. 2E), highlighting the pivotal role of CCL2 in attracting monocytes by astrocyte stimulated with anti-AQP4 Ab (control vs GK89; p = 0.041, GK89 vs GK89 + anti-CCL2 antibody; p = 0.028).

### Phenotypic change of monocyte by AQP4-Ab conditioned medium

**Figure 3:**
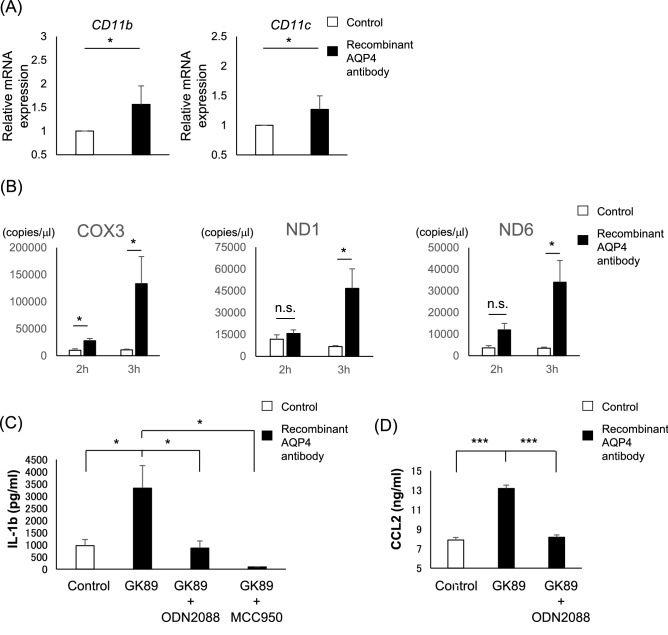
The GK89-treated supernatant of human astrocytes induces monocyte activation. **(A)** Gene expression of CD11b and CD11c in monocytes stimulated by GK89-treated the supernatant of human astrocytes. Values are normalized to GAPDH. **(B)** The GK89-treated supernatant of human astrocytes show chronological increase of extracellular mtDNA after antibody exposure. **(C, D)** CD14^+^ monocytes exposed to mtDNA purified from GK89-treated supernatant of human astrocytes secrete a significant amount of IL1β **(C)** and CCL2 **(D)** release, which is efficiently inhibited by MCC950 and ODN2088. Error bars indicate SEM. Three or more experiments are performed in each condition. *p < 0.05, ***p < 0.001.

In addition to the enhanced recruitment, recruited monocytes can also be activated in the inflammatory milieu. Therefore, we further assessed for the phenotypic change of human monocyte exposed to GK89-treated ACM. CD86 expression level was enhanced among the activation markers, such as CCR2, HLADR, and CD80 (Supplementary Fig. S1). Quantitative PCR analysis also showed that CD14^+^ monocytes exposed to GK89-treated ACM exhibit enhanced expression of adhesion molecules such as CD11b (p = 0.049) and CD11c (p = 0.049) (Fig. 3A).

A previous report showed that NMOSD-immunoglobulin induce astrocytic cytotoxicity and further induce the release of extracellular mtDNA^9^. Consistent with these observations, ACM showed chronological increase of extracellular mtDNA concentration after GK89-Ab exposure (COX3_2h/control; 10,016 ± 2,821, GK89; 27,866 ± 4,266, p = 0.049), (COX3_3h/control; 10,925 ± 1,432, GK89 133,367 ± 50,250, p = 0.049), (ND1_3h/control; 6,684 ± 773, GK89; 46,670 ± 13,474, p = 0.049), (ND6_3h/control; 3,515 ± 453, GK89; 33,985 ± 10,119, p = 0.049) (Fig. 3B). In addition, mtDNA treatment of CD14^+^ monocytes led to IL1β release (control; 963.76 ± 252.37, GK89; 3,329.44 ± 925.53, p = 0.049), which was significantly inhibited by MCC950 (inflammasome inhibitor) (GK89 + MCC950; 97.32 ± 4.69, p = 0.049) and ODN2088 (TLR9 inhibitor) (GK89 + ODN2088; 869.02 ± 295.74; p = 0.049) (Fig. 3C). Moreover, monocytes treated with mtDNA extracted from ACM secreted a significant amount of CCL2 in a TLR9-dependent manner (control; 7.90 ± 0.26, GK89; 13.18 ± 0.34, GK89 + ODN2088; 8.16 ± 0.25, control vs GK89; p = 0.00074, GK89 vs GK89 + ODN2088; p = 0.00092) (Fig. 3D). These results suggest that GK89-treated ACM change monocyte activation status, represented by enhanced expression of co-stimulatory and adhesion molecules, and by production of IL-1β and CCL2.

### The number of CSF monocyte correlates with mitochondrial DNA levels in NMOSD patients

**Figure 4:**
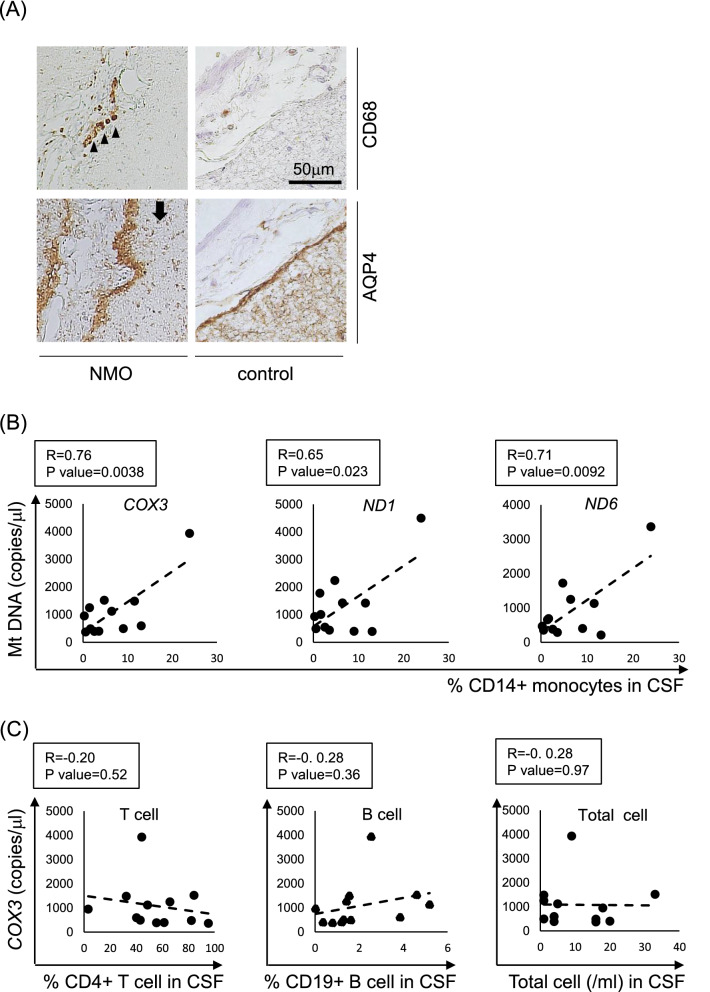
Preferential recruitment of monocyte is observed at the active lesions of NMOSD and the CSF monocyte count correlates with mitochondrial DNA levels in NMOSD patients. **(A)** In the NMOSD brain parenchyma, CD68-positive monocytes are detected at the subarachnoid space (arrow heads) where the loss of AQP4 staining is observed (arrow) (left column). In control brain, CD68-positive monocytes are absent where the AQP4 expression is preserved (right column). **(B)** MtDNA gene levels show positive correlation with the percentage of CD14^+^monocytes in NMOSD patients’ CSF. **(C)** COX3 gene expression level does not show significant correlation with the percentage of CD4^+^ T cells, CD19^+^ B cells, or the number of total cells in the NMOSD patients’ CSF. Scale bar: 50 µm

An immunohistochemical study exhibited that CD68-positive monocytes were detected at the subarachnoid space in NMOSD, where the loss of AQP4 staining was observed (Fig. 4A (left column)). CD68-positive monocytes were rarely observed in the control group (Fig. 4A (right column)). These results highlight the active monocyte recruitment near the active lesions of NMOSD, where AQP4 expression is diminished.

As astrocytes exposed to anti-AQP4 Abs were shown to preferentially recruit monocytes in vitro, we investigated whether CSF monocyte counts correlate with astrocyte damage in NMOSD patients. For this purpose, we investigated the correlation between CSF mtDNA levels and various immune cell subsets in CSF and we confirmed the positive correlation between mtDNA and CD14^+^ monocytes in the CSF of NOMSD patients. For other cell subtypes, we didn’t find any significant correlation (Fig. 4B, C).

These results suggest that CD14^+^ monocytes are preferentially recruited within the CNS after astrocytic damage by the release of mtDNA in NMOSD patients.

## Discussion

In this study, we showed that mtDNA released from astrocyte stimulated by anti-AQP4 Ab serve as a molecular bridge of innate immunity, by enhancing monocyte activation and recruitment via CCL2 in NMOSD pathogenesis.

As for the role of monocytes in the acceleration of lesion formation in NMOSD, a previous report showed the reduction in the lesion size after macrophage depletion with clondronate liposomes in the NMOSD rat model^7^. In this study, we revealed that CCL2 generated from astrocytes had a critical role in recruiting monocytes. Consistently, previous literature showed that anti-AQP4 Ab stimulated astrocytes to release CCL2^11^ and the ablation of CCL2 released from astrocytes reduced the recruitment of peripheral monocytes to CNS and dampened axonal loss and neurological deficit^12^. Another study also reported that accumulation of lactosylceramide (LacCer) via β-1,4-galactosyltransferase6 (B4GALT6) promoted CCL2 production in astrocyte and B4GALT6 was actually upregulated in astrocytes expressing CCL2 in MS lesions^13^. In addition, previous reports showed that CCL2, CCL3 and CCL4, the chemokines for attracting monocytes, were elevated within the CSF of patients with NMOSD^14^. Thus, it is plausible that for the massive infiltration of monocytes in the lesions of NMOSD, CCL2 serves as the critical factor to enhance the innate immune response.

On the other hand, our recent report showed that mtDNA, one of the damage-associated molecular patterns (DAMPs), in CSF was elevated in NMOSD and mtDNA concentration was reduced after immunotherapy^9^. These observations indicated that mtDNA might be a critical factor for forming NMOSD pathology. So we hypothesized that mtDNA released from damaged astrocytes might be involved in monocyte activation in NMOSD. Actually, in this study, we showed that monocytes exposed to ACM containing abundant mtDNA facilitated expression of CD86, CD11b and CD11c, and mtDNA further accelerated monocytes to secrete IL-1β in TLR9 and inflammasome-dependent manners. IL-1β not only activates leukocytes^15,16^ but also makes the blood–brain barrier leaky enhancing monocyte migration^17^ and IL-1β are shown to have essential roles in forming NMOSD lesions in a rat model^18^. Furthermore, in this study we elucidated the inflammatory loop of monocyte recruitment in NMOSD, where monocytes themselves enhanced the production of monocyte-recruiting chemokine after being exposed to mtDNA. Together, these observations suggest that astrocytes not only play an essential role in recruiting monocytes, but they also promote the activation of monocytes, and mtDNA serve as a critical factor in enhancing the inflammatory conditions.

Importantly, we observed that CD68-positive monocytes were preferentially recruited at the subarachnoid space in the active lesion of NMOSD. Three pathways, the entries through circumventricular organs, subarachnoid space and parenchymal vessel, were previously reported to have crucial roles in immune cell invasion to the CNS^19^, and monocyte infiltration through the CNS surface is observed at the early phase in experimental autoimmune encephalomyelitis (EAE)^20^. Thus, our results suggest the importance of the subarachnoid pathway in NMOSD pathology. Moreover, anti-AQP4 antibody-induced astrocyte loss is reported at the pial glial limitans, and microglial revitalization is known to take place at the pila surface of the CNS in NMOSD^21^. This observation is consistent with our results that the degree of monocyte migration correlates with the concentration of mtDNA, the astrocyte destruction marker in NMOSD CSF.

A previous report showed the elevation of DAMPs, such as HMGB1, in NMOSD CSF but did not reveal the association between the increase of DAMPs and NMOSD pathogenesis^22^. By demonstrating that mtDNA concentration within the CSF show positive correlation with monocyte population in NMOSD patients, the clinical relevance of mtDNA as an initial trigger to enhance innate immunity in NMOSD is suggested in our study. A previous report elucidated the amount of CCL2 correlated with the copy number of mtDNA in the CSF of HIV CNS inflammation^23^. In this sense, our observation indicates that CNS damage could be directly related to monocyte migration via pathogenic mtDNA release.

**Figure 5:**
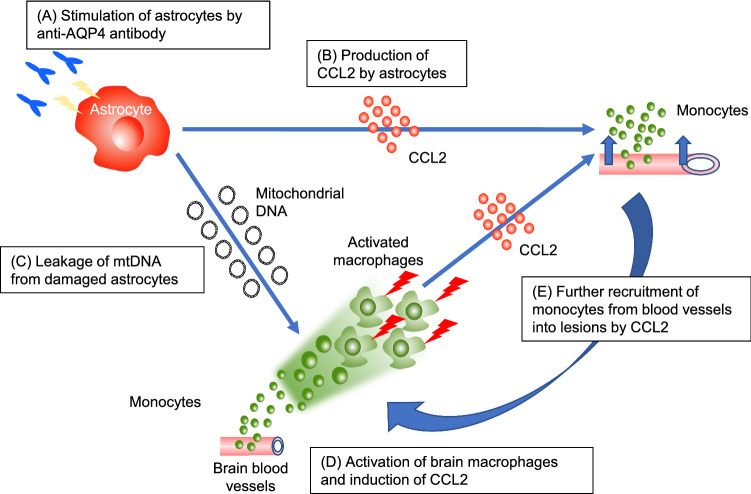
The scheme shows mtDNA serve as a molecular bridge of the inflammatory loop in NMOSD by activating and attracting monocytes from peripheral blood. **(A)** Anti-AQP4 Ab stimulate astrocytes. **(B)** CCL2 is produced by the stimulated astrocytes. **(C)** MtDNA is released from the astrocytes damaged by anti-AQP4 Ab. **(D)** MtDNA activates macrophages infiltrating into the parenchyma and induces the production of CCL2. **(E)** CCL2 recruits the monocytes from blood vessels into lesions, generating the inflammatory loop of monocyte recruitment and activation.

Taken together, mtDNA which was released from astrocytes serve as a molecular bridge of innate immunity in NMOSD by activating and attracting monocytes from peripheral blood, further accelerating the inflammatory loop so as to exacerbate CNS damage (Fig. 5).

## Methods

### Patient information and CSF sample collection

CSF samples were obtained by patients with NMOSD positive for anti-AQP4 antibody, fulfilling the 2015 NMOSD diagnostic criteria^24^. Each sample was collected at the relapse phase, defined as appearance of new neurological symptoms lasting more than 24 h. Informed consent was obtained from each patient. Human CSF was immediately centrifuged at 400 *g* for 10 min after lumbar puncture and stocked at − 80 °C. All of the patients’ characteristics were shown in Table 1.

**Table 1:** Clinical characteristics and CSF data of patients with NMOSD.

	Sex	Age	Duration from relapse	Laboratory data in CSF				Laboratory data in blood		Lesions in MRI analysis		
				Total cell (lymphocyte) (1/µl)	Protein (mg/dl)	IgG index	OCB	Anti-AQP4 antibody	Other antibody	Spinal cord	Brain	Optic nerve
NMO_1	F	47	3 months	4 (4)	64	0.43	−	75 <	–	C2–C5	Multiple	−
NMO_2	F	71	3 months	16 (14)	46	0.49	−	75 <	–	C1–C6	Multiple	−
NMO_3	F	30	4 days	16 (14)	59	0.65	+	75 <	ANA Anti-SS-A	Th1-8	–	+
NMO_4	F	50	21 days	33 (26)	70	Not measured	−	75 <	–	C1-3 C7-Th8	–	+
NMO_5	F	37	5 days	18 (13)	47	Not measured	−	+	Anti-SS-A Anti-SS-B	C2-4	A few	−
NMO_6	F	43	7 days	1 (1)	32	0.46	−	+	Anti-SS-A	C3-Th6	Brain stem	−
NMO_7	F	55	7 days	9 (7)	36	0.54	−	75 <	–	Th3	A few	+
NMO_8	F	51	58 days	1 (1)	29	0.51	Not measured	5.5	–	–	–	+
NMO_9	F	67	18 days	20 (15)	85	0.478	−	+	–	C2-C6	A few	−
NMO_10	F	41	30 days	5 (5)	38	0.56	−	+	ANA	Th3-4	–	−
NMO_12	F	55	15 days	1 (1)	35	0.41	−	15.3	–	Th6-8	Multiple	−
NMO_13	F	33	2 days	4 (4)	31	0.65	+	75 <	ANA Anti-SS-A	Th1-8	–	+

*NMO* neuromyelitis optica, *CSF* cerebrospinal fluid, *lympho* lymphocyte, *OCB* oligo clonal band, *ANA* anti-nuclear antibody.

### Generation of monoclonal antibodies by single-cell sorting

After lumbar puncture, CSF samples were immediately centrifuged at 400 *g* for 10 min. Cells were stained with PECy7-conjugated CD3 (TONBO biosciences, diluted 1:200), PB-conjugated CD14 (BD pharmingen, diluted 1:200), APC-conjugated CD19 (eBioscience, diluted 1:50) and PE-conjugated CD138 (BD parmingen, diluted 1:50) and single-cell sorting was performed on Aria flow cytometer (BD biosciences), and CD3^-^ CD19^int^ CD138^+^ plasmablasts were isolated. The collected cells were lysed with NP40 and directly reverse-transcripted with ReverTra Ace (TOYOBO), according to the instruction protocol. V regions of IgH, and either IgK or Igλ were amplified by nested PCR from the reconstructed cDNA^25–27^. The generated V regions were then ligated into p3XFLAG-CMV-14 expression vectors (Sigma-Aldrich), which harbored constant regions of human IgG1, Igκ or Igλ. After the plasmids were transfected into HEK293 cells, antibodies produced in the supernatant were purified with protein G sepharose (GE Healthcare). The IgG concentration of each sample was determined by ELISA (capture antibody; SouthernBiotech, alkaline phosphatase conjugated detection antibody; SouthernBiotech, p-Nitrophenyl phosphate; SIGMA). And for mass production, using EF1a-based expression vector (kindly provided by Chugai Pharmaceutical Co.), we requested Thermo Fisher Scientific to generate and purify recombinant antibodies.

### Identification of somatic hypermutations

The sequenced results of V regions in each clone were analyzed by IMGT/V-QUEST, and somatic hypermutations were identified by comparing them to germline DNA sequences.

### Culture of HEK cells

HEK293 cells transfected with or without M23-human AQP4 expression plasmids (GeneCopoeia, Rockville, MD) were cultured in Dulbecco’s Modified Eagle’s Medium, containing 10% fetal bovine serum and 1% penicillin–streptomycin, as previous described.

### Immunocytochemistry

One day after cells were seeded on 16-well chamber slides, 2 µg/ml of generated recombinant Abs were incubated for 30 min on ice, and 1:200 FITC-conjugated anti-human IgG (Southern Biotech) was used as a secondary antibody for 30 min on ice after three washes. Finally these cells were fixed with 4% paraformaldehyde (Nakarai) for 10 min and the cells were observed by BZ-X700 fluorescence microscope (Keyence).

### Binding assay of recombinant Abs

2 µg/ml of recombinant Abs were incubated with AQP4-expressing HEK293 cells for 30 min on ice, followed by 5 µg/ml FITC-conjugated anti-human IgG (Southern Biotech) as a secondary antibody for 20 min on ice. The mean fluorescence intensity (MFI) of each sample was analyzed with Canto II flow cytometer (BD biosciences).

### LDH release assay

AQP4-expressing HEK293 cells were treated for 4 h with 2.5 µg/ml recombinant Abs or isotype control (BioLegend) in the presence of 2% rabbit serum (MP Biomedicals). Released lactate dehydrogenase (LDH) was measured with a LDH assay kit (DOJINDO, Japan).

### Culture of human astrocyte

Human primary astrocytes were purchased from ScienCell Research Laboratories (Carlsbad, CA), and were cultured in a supplemented astrocyte medium (ScienCell Research Laboratories) according to the supplier’s instructions.

### Quantitative PCR of human astrocytes

Human astrocytes treated by GK89 or isotype control Abs for 4 h were dissolved to isogenII. 60 ng RNA extracted according to the instruction protocol was reverse-transcripted with SuperScript VILO (thermos Fisher Scientific) as follows: 25 °C for 10 min, 42 °C for 60 min and 85 °C for 5 min.

cDNA corresponding to 1 ng RNA was measured with qPCR. The reaction conditions for CCL2 (forward 5′-ACTCTCGCCTCCAGCATGAA-3′, reverse 5′-TTGATTGCATCTGGCTGAGC-3′), CCL4 (forward 5′-CGCCTGCTGCTTTTCTTACAC-3′, reverse 5′-GGTTTGGAATACCACAGCTGG-3′), CXCL8 (forward 5′-CCTTCCTGATTTCTGCAGCTCT-3′, reverse 5′-GGTGGAAAGGTTTGGAGTATGTCT-3′) and β2-microglobulin (β2-M_1; forward 5′-GCTATCCAGCGTACTCCAAAGATTC-3′, reverse 5′-CAACTTCAATGTCGGATGGATGA-3′) were as follows: 95 °C for 20 s and then 40 cycles of 95 °C for 1 s and 60 °C for 20 s ^28^. The housekeeping β2-microglobulin gene was selected for normalization. The reaction conditions for CCL7 (forward 5′-CCTGGACAAGAAAACCCAAA-3′, reverse 5′-TTCAAAACCCACCAAAATCC-3′), CCL20 (forward 5′-GCAAGCAACTTTGACTGCTG-3′, reverse 5′-CAAGTCCAGTGAGGCACAAA-3′) and CX3CL1 (forward 5′-GAGTGGGTCCAATGCACTTT-3′, reverse 5′-CACAGACGTTGGTGATGAGG-3′) and glyceraldehyde-3-phosphate dehydrogenase (GAPDH; forward 5′-ATCACCATCTTCCAGGAG-3′, reverse 5′-ATCGACTGTGGTCATGAG-3′) were as follows: 95 °C for 10 min and then 40 cycles of 94 °C for 10 min, 57 °C for 30 s and 72 °C or 30 s ^29^. The housekeeping GAPDH gene was selected for normalization. The reaction conditions for CCL3 (forward 5′-AGCTGACTACTTTGAGACGAGCA-3′, reverse 5′-CGGCTTCGCTTGGTTAGGA-3′), CCL5 (forward 5′-GACACCACACCCTGCTGCT-3′, reverse 5′-TACTCCTTGATGTGGGCACG-3′) and β2-microglobulin (β2-M_2; forward 5′-CTCCGTGGCCTTAGCTGTG-3′, reverse 5′-TTTGGAGTACGCTGGATAGCCT-3′) were as follows: 50 °C for 2 min and 95 °C for 10 min and then 40 cycles of 95 °C for 15 s and 60 °C for 1 min^30^. The housekeeping β2-microglobulin gene was selected for normalization. Results were analyzed by Applied Biosystems 7900HT (Carlsbad, CA).

### Preparation of ACM

Primary human astrocytes were treated with 50 µg/ml GK89 or isotype control Abs (BioLegend) in the presence of 10% human serum (Sigma-Aldrich), and the obtained supernatant was immediately centrifuged at 400 *g* for 10 min after 2 h and 3 h exposure respectively, and stocked at − 80 °C.

### Migration assay

2 × 10^5^ PBMC in RPIM without FCS were placed to the upper side of 5 µm-pore two chamber wells (24wells) (Corning), and ACM was added in the lower well. After incubation for 2 h, the migrated cells were stained for PECy7-conjugated CD4 (Biolegend, diluted 1:50), APC-conjugated CD8 (Miltenyi Biotec, diluted 1:50), APC-conjugated CD14 (Bioledend, diluted 1:50) and APC/Cy7-conjugated CD19 (Biolegend, diluted 1:100) after FcR block for 15 min (Miltenyl Biotec, diluted 1:100) and analyzed by Canto II flow cytometer (BD biosciences).

For the inhibitory assay of CCL2, 5 × 10^4^ THP1 monocytes in RPMI without FCS was placed in the upper chamber of 8 μm-pore two chamber wells (24 wells) (Corning). After 30 min incubation with ACM and anti-CCL2 neutralizing antibody (R&D) at room temperature, the number of migrated cells for 2 h was counted with trypan blue (Gibco).

### Quantitative PCR analysis of human monocytes

Human monocytes were isolated with CD14 magnetic beads (Miltenyi Biotec) from human PBMCs, and 1 × 10^5^ monocytes were seeded on 96-well plates in the presence of ACM for 24 h. After the incubation, monocytes were collected and stored in IsogenII. The total RNA was extracted and directly reverse-transcribed with SuperScript VILO (Thermo Fisher Scientific) according to the manufacturer’s protocol. Quantitative PCR analysis was performed with TaqMan probe against CD11b, CD11c and GAPDH (as internal control) (Applied Biosystems, USA; # Hs00167304, # Hs00174217 and # Hs03929097, respectively). Results were analyzed by Applied Biosystems 7900HT (Carlsbad, CA).

### Surface markers of human monocytes

Human monocytes isolated with CD14 magnetic beads (Miltenyi Biotec) from human PBMCs were exposed for 5 days in the presence of ACM. Cells were then collected and stained with APC-conjugated CCR2 (Biolegend, diluted 1:50), PE-conjugated CD14 (Biolegend, diluted 1:100), APC-conjugated HLADR (Biolegend, diluted 1:50), FITC-conjugated CD80 (BD pharmingen, diluted 1:50) and FITC-conjugated CD86 (BD pharmingen, diluted 1:50) after the FcR block for 15 min (Miltenyl Biotec, diluted 1:100). In high CD14 expressing cells, we examined the intensity with the other antibody. The mean fluorescence intensity (MFI) of each sample was analyzed with Canto II flow cytometer (BD biosciences).

### Quantitative PCR of mtDNA

DNA fraction in 100 μl ACM or human CSF was purified with a DNA extractor SP kit (Wako, Osaka, Japan), and separated DNA was reconstituted in 20 μl DDW. Purified DNA fraction was amplified with SYBR Green (Takara Bio). The used primers were as follows: mtDNA gene encoding cytochrome C oxidase 3 (COX3; forward 5′-ATGACCCACCAATCACATGC-3′, reverse 5′-ATCACATGGCTAGGCCGGA-3′), NADH dehydrogenase1 (ND1; forward 5′-ATACCCATGGCCAACCTCCT-3′, reverse 5′-GGGCCTTTGCGTAGTTGTAT-3′), and NADH dehydrogenase 6 (ND6; forward 5′-CCCCTGACCCCCATGCCTCA-3′, reverse 5′-GCGGTGTGGTCGGGTGTGTT-3′). Standard curves were made according to serial dilution of a plasmid including each sequence, prepared by Nihon Gene Research Laboratories (Sendai, Japan). Results were analyzed by Applied Biosystems 7900HT (Carlsbad, CA).

### IL-1β assay

1 × 10^5^ human monocytes were isolated with CD14 magnetic beads (Miltenyi Biotec) from human PBMCs. CD14^+^ monocytes were treated with mtDNA extracted from 80 µl ACM for 24 h after being pretreated for 1 h with either 10 μM MCC950 (inflammasome inhibitor), 2 μM ODN2088 (TLR9 inhibitor) or only medium respectively. On the following day, the plate was centrifuged at 400 *g* for 10 min, and the supernatants were collected. The IL-1β in supernatants was measured using ELISA kit (abcam) according to the manufacturer’s instruction.

### CCL2 assay

1 × 10^5^ CD14^+^ human monocytes from human PBMCs were treated with mtDNA extracted from 20 µl ACM after pretreatment with 2 μM ODN2088 for 1 h. CCL2 in the supernatants was analyzed with an ELISA kit (R&D) according to the manufacturer’s instruction.

### Immunohistochemistry

Brain blocks from six autopsy cases (3 NMOSD and 3 disease controls) were used. NMOSD samples included a 67-year-old (yo) female, a 44-yo female and a 80-yo female. The disease control samples contained a 63-yo male (MS), a 70-yo male (stroke) and a 56-yo male (chronic inflammatory demyelinating polyneuropathy). The formalin-fixed paraffin-embedded blocks were cut into 5 mm thick sections and were de-waxed. The sections were stained with ant-AQP4 rabbit antibody (1:1,000, Proteintech) or the anti-CD68 mouse antibody (undiluted, DAKO), followed by a secondary antibody conjugated with HRP (DAKO). Positive staining was detected by ImmPACT HRP substrate (Funakoshi).

### CSF analysis of monocytes

CSF derived from 12 NMOSD patients were immediately centrifuged at 400 *g* for 10 min after lumbar puncture, and treated with the FcR blocking reagent (Miltenyi Biotec) for 15 min on ice. The cells were then stained with PE-conjugated CD4 (BioLegend), FITC-conjugated CD19 (BD Biosciences), and APC-conjugated CD14 antibodies (BioLegend) for 30 min on ice, and analyzed by Canto II flow cytometer (BD biosciences).

### Statistical analysis

P values were calculated by the Mann–Whitney U tests (two-tailed) or T-test using SPSS software. Data are represented as average ± SEM. Statistical significance is reported as not significant; (n.s.), *; p < 0.05, **; p < 0.01 and ***; p < 0.001.

### Ethics approval and consent to participate

All of the methods and experiments were conducted in accordance with the revised Declaration of Helsinki and Good Clinical Practice guidelines and was approved by the ethics committee of Osaka University Hospital (permit number 12091-6). We confirmed that informed consent was obtained by all participants or their legal representative.

## Supplementary information

Supplementary Information.

## Data Availability

The datasets used and analyzed during the current study are available from the corresponding author on reasonable request.
