# Supplementary material for: Mitochondrial DNA enhance innate immune responses in neuromyelitis optica by monocyte recruitment and activation
Source: Sci Rep. 2020 Aug 6;10:13274. doi: 10.1038/s41598-020-70203-x (PMC7414017; doi:10.1038/s41598-020-70203-x)
Supplement: Supplementary file 1 — Supplementary Information. [file 41598_2020_70203_MOESM1_ESM.pdf]

Supplementary Information (Supplementary Figure)

Title; Mitochondrial DNA enhance innate immune responses in neuromyelitis optica by monocyte recruitment and activation

Authors

Mikito Shimizu, MD, PhD[1,+], Tatsusada Okuno, MD, PhD[1,*,+], Makoto Kinoshita, MD, PhD[1,+], Hisae Sumi, MD, PhD[1], Harutoshi Fujimura, MD, PhD[2], Kazuya Yamashita, MD, PhD[1], Tomoyuki Sugimoto, PhD[3], Shuhei Sakakibara, PhD[4], Kaori Sakakibara, PhD[1], Toru Koda, MD, PhD[1], Satoru Tada, MD, PhD[1], Teruyuki Ishikura, MD[1], Hisashi Murata, MD[1], Shohei Beppu, MD[1], Naoyuki Shiraishi, MD[1], Yasuko Sugiyama, MD[1], Yuji Nakatsuji, MD, PhD[5], Atsushi Kumanogoh, MD, PhD[6], Hideki Mochizuki, MD, PhD[1],

Affiliation

[1]Department of Neurology, Osaka University Graduate School of Medicine, 2-2, Yamadaoka, Suita, Osaka, 565-0871, Japan
[2]Department of Neurology, Osaka-Toneyama National Medical Center, 5-1-1, Toneyama, Toyonaka, Osaka, 560-8552, Japan
[3]Graduate School of Data Science, Shiga University, 1-1-1, Baba, Hikone, Shiga, 522-8522, Japan
[4]Laboratory of Immune Regulation, Immunology Frontier Research Center, Osaka University, 3-1, Yamadaoka, Suita, Osaka, 565-0871, Japan
[5]Department of Neurology, Faculty of Medicine, University of Toyama, 2630, Sugitani, Toyama, 930-0194, Japan
[6]Department of Respiratory Medicine and Clinical Immunology, Osaka University Graduate School of Medicine, 2-2, Yamadaoka, Suita, Osaka, 565-0871, Japan

*Correspondence Authors e-mail; okuno@neurol.med.osaka-u.ac.jp,
+these authors contributed equally to this work

Supplementary Figure S1

Title; FACS analysis of monocyte stimulation to ACM

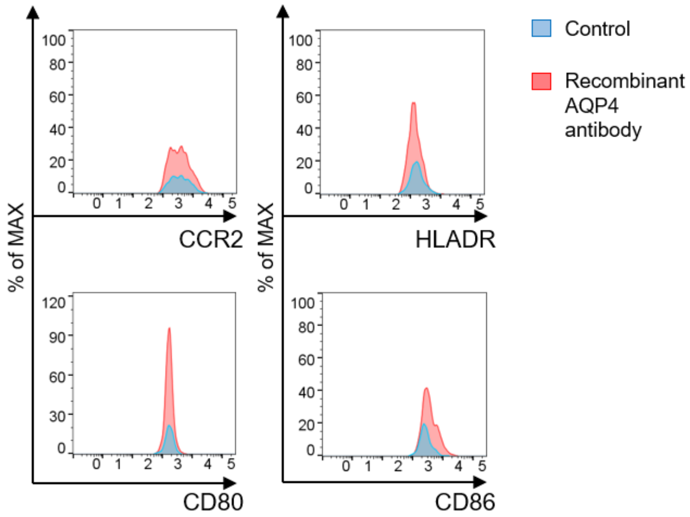

Legend; Monocytes exposed to GK89-treated supernatant of human astrocytes show an enhanced expression level of CD86, but not CCR2, HLADR and CD80.
